# T‐cell blast crisis of chronic myelogenous leukemia presented with coexisting p210 and p190 BCR‐ABL transcripts and t(10;11)(q11;p15)

**DOI:** 10.1002/jcla.23241

**Published:** 2020-02-13

**Authors:** Ting Xia, Yuchao Yang, Guoxia Li, Jianmei Chang, Jianlan Li, Fanggang Ren, Weixiao Ren, Hongwei Wang, Zhifang Xu

**Affiliations:** ^1^ The Haematology Department The Second Hospital of Shanxi Medical University Taiyuan Shanxi China

**Keywords:** BCR‐ABL, blast crisis, chronic myelogenous leukemia, T‐Lineage acute lymphoblastic leukemia

## Abstract

**Background:**

Blast transformation of chronic myelogenous leukemia (CML) to T lymphoblastic lymphoma/acute lymphoblastic leukemia (T‐LBL/ALL) is rare, and the molecular mechanism is still unclear.

**Case report:**

A 28‐year‐old woman who developed T‐ALL with coexpressing both p210 and p190 BCR‐ABL transcripts five years after the initial diagnosis of CML in chronic phase. The proliferation of bone marrow was extremely active with blast cells over 20%. Chromosome analysis revealed t(9;22)(q34;q11) and t(10;11)(q25;p15). Flow immunophenotyping showed that blasts expressed CD4, CD7, CD11b, CD38, CD34, CD33, and cCD3.

**Conclusion:**

It is the first T‐cell blast of CML case with coexisting p210 and p190 as well as additional chromosome translocations. Through review this case and previous reports, we will reveal that CML patients with T‐lymphocyte transformation depend on potential molecular and pathological mechanism.

## 1 INTRODUCTION

Chronic myelogenous leukemia (CML) is a common myeloproliferative disorder originating from clone abnormal proliferation of hematopoietic stem cells. The natural course of CML consists of three stages: chronic phase (CP), accelerated phase (AP), and blast crisis (BC). CML‐BC, the late stage of CML, usually means the worsening of malignancy and poor prognosis. In the CML‐BC, the majority progressed to acute myeloid leukemia (AML) and the rest to lymphoid blast. Almost all patients with lymphocytic blast were B‐ALL and rarely involved in T‐lineage.^1^

The BCR‐ABL fusion gene, derived from chromosomal translocation t(9;22)(q34;q11.2), is considered to be a molecular marker of CML. According to the different breakpoint site, the BCR‐ABL transcripts are divided into three subtypes of M‐bcr, m‐bcr, and μ‐bcr, encoding protein p210, p190, and p230, respectively. M‐bcr (p210) is expressed in 95% CML and a few of Ph + ALL, m‐bcr (p190) is almost all expressed in Ph + ALL, and μ‐bcr (p230) is rare, mainly present in chronic neutrophilic leukemia. CML with coexpressing p210 and p190 is rare, which may be associated with progress of ALL. In addition to t(9;22), CML‐BC is usually accompanied by additional chromosomal abnormalities (ACAs).^2^

It is not entirely clear about the molecular mechanism of CML transformation into T‐LBL/ALL. Studies showed that clone evolution of BCR‐ABL fusion gene and ACAs may play important roles in blastic transformation of CML.^3^ Here, we discussed the potential molecular mechanisms of CML to T‐cell blast by reviewing an unusual case of coexpressing p210 and p190 as well as aberration of chromosome 11p15.

## 2 CASE DESCRIPTION

**Figure 1:**
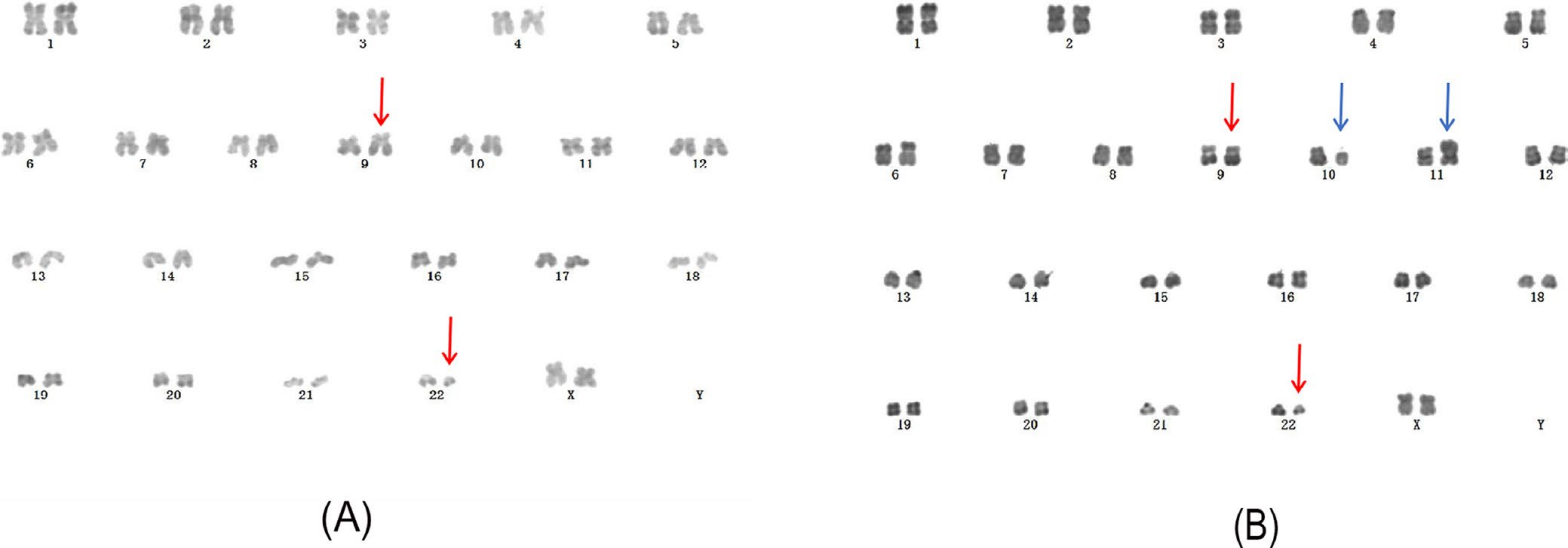
G‐banding karyotype of bone marrow in chronic phase (A) and blast crisis (B). Red arrow: t(9;22)(q34;q11.2), blue arrow: t(10;11)(q11;p15)

**Figure 2:**
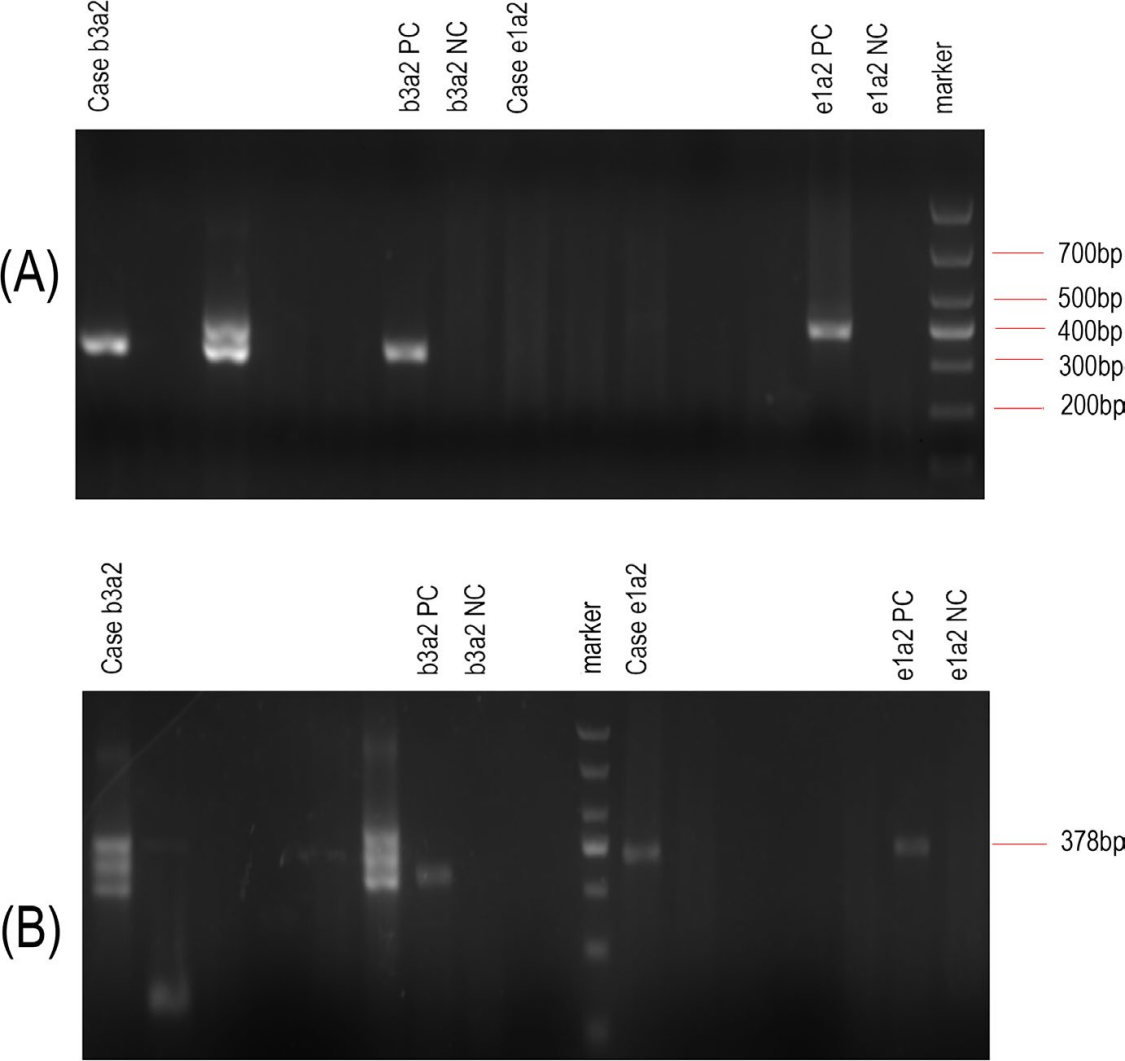
The agarose gel electrophoresis picture of BCR‐ABL fusion gene at diagnosis (A) and in blast crisis (B). The case is from the patient sample, and b3a2 (p210) is 319bp, while e1a2 (p190) is 378bp. PC, positive control; NC, negative control; the molecular weight marker is shown at right end (A) and middle (B), respectively. Unlabeled lanes are BCR‐ABL fusion gene amplification bands from other patients

**Figure 3:**
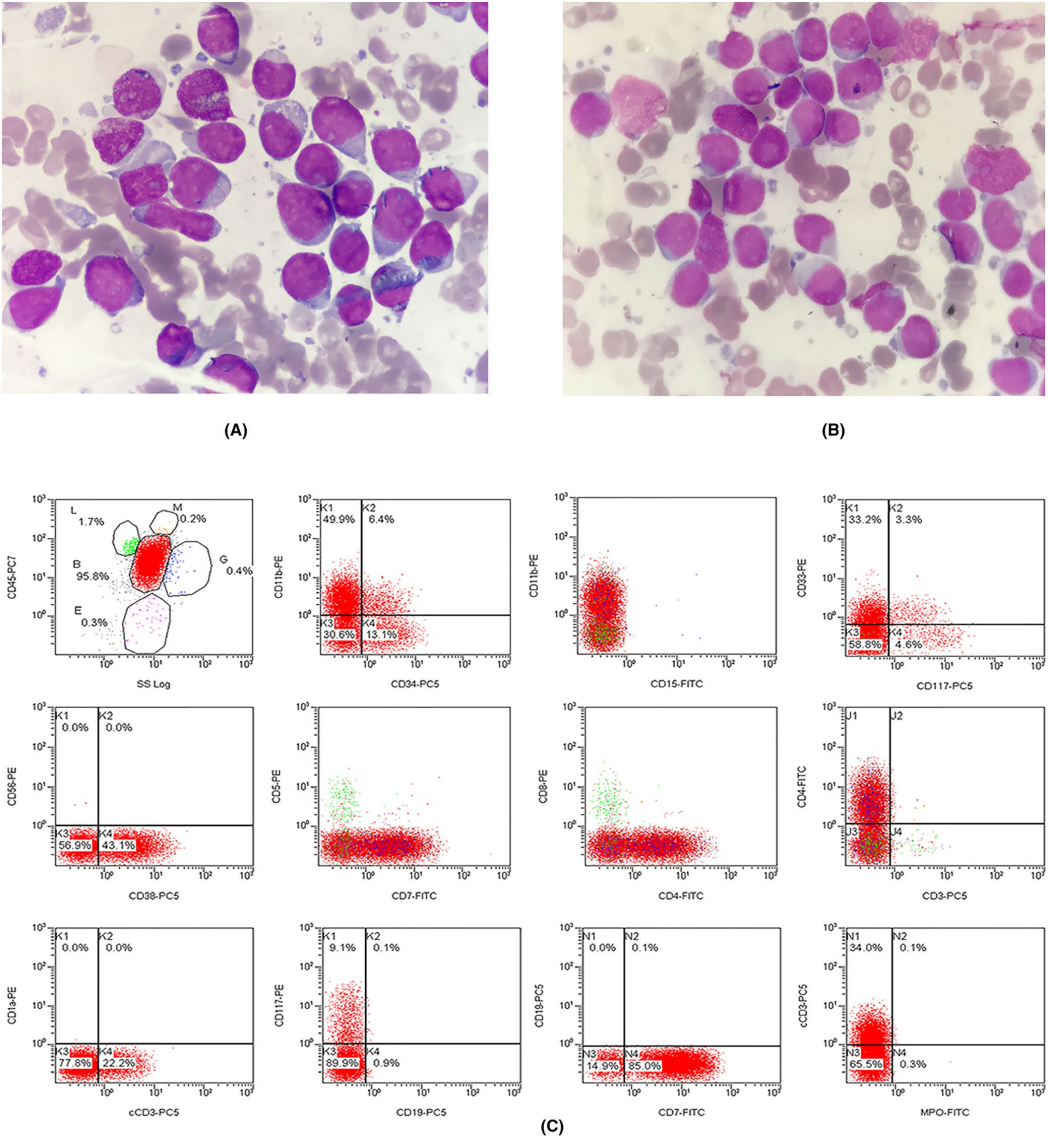
(A) and (B), Bone marrow morphology with lymphatic hyperplasia in blast crisis in two microscopic fields (Wright‐Giemsa, 1000×). (C), Flow immunophenotypes of bone marrow in blast crisis

A 24‐year‐old woman was initially identified as CML for bone marrow (BM) smear image, cytogenetic karyotype 46, XX, t(9;22)(q34;q11) (Figure 1A), and the BCR/ABL (p210) fusion transcript (Figure 2A) by reverse transcriptase‐polymerase chain reaction (RT‐PCR). She was treated with hydroxyurea (the specific dose is unknown). Three years later, the patient presented with masses in the bilateral mandibular without fever. BM smear and karyotype indicated CML was in chronic phase. But p210 was still replicated with 163 copies/95117 ABL copies. She was treated with imatinib 400 mg/d. Five years later, she was re‐admitted to our hospital for multiple neck masses and progressive swelling with fever and sore throat. Physical examination: moderate anemia appearance, body temperature 38.8 degrees, a number of swollen lymph nodes on bilateral submandibular, submental, and left cervical posterior, some fused into lumps, up to 4 cm × 5 cm, hard, poor mobility, and nontender. Laboratory tests: WBC: 40.00 × 10^9^/L, HGB: 75.0 g/L, PLT: 9.0 × 10^9^/L, lymphocyte: 80.30%, and neutrophil: 1.9%. BM showed marked hyperplasia with more than 20% blasts (Figure 3A,B). Karyotype: 46, XX, t(9;22)(q34;q11)[20]/46, XX, t(10;11)(q11;p15)[20] (Figure 1B). BCR/ABL fusion genes b3a2 and e1a2 were coexisted (Figure 2B). Flow cytometry of BM showed blasts accounted for 95.8% and expressed CD4, CD7, CD11b, and CD38, partial cytoplasmic (cy) CD3, CD34, and CD33, while lacking CD19, CD11a, cCD79a, and cMPO (Figure 3C). CML‐BC involving T cell was considered. In view of ABL kinase domain E255K mutation, dashatinib was administered to her with a significant decrease in WBC (as shown in Table 1). She stopped treatment and died (2 months after blast crisis) by follow‐up.

**Table 1:** Changes of blood parameters in blast crisis

Date	WBC	HB	PLT	LY#	MO#	NE#	BA#	LY%	MO%	NE%	BA%
2016.5.17	40.00	75.00	9.00	32.10	7.32	0.78	0.00	80.30	18.30	1.90	0.00
2016.5.18	32.17	65.00	21.00	25.37	6.16	0.60	0.02	78.80	19.10	2.00	0.10
2016.5.19	67.01	68.00	22.00	65.29	0.65	0.00	0.01	97.40	1.00	0.00	0.00
2016.5.20	94.15	69.00	50.00	73.43	0.05	0.00	0.18	78.00	0.10	0.00	0.20
2016.5.21	127.65	66.00	26.00	102.72	0.29	0.00	0.20	80.50	0.20	0.00	0.20
2016.5.22	170.70§	70.60	21.80	11.16	72.28	87.26	0.00	6.54	42.34	51.12	0.00
2016.5.23	140.21§	59.00	12.00	113.64	16.19	0.00	0.21	81.10	11.50	0.00	0.10
2016.5.24	33.74§	57.00	6.00	30.96	1.66	0.00	0.01	91.80	4.90	0.00	0.00
2016.5.25	13.16§	60.00	6.00	12.65	0.46	0.05	0.00	96.14	3.54	0.44	0.04
2016.5.26	9.94§	88.00	9.00	7.99	1.58	0.00	0.00	80.40	15.90	0.00	0.00

Abbreviations: %, percentage; BA, basophil; HB, hemoglobin; LY, lymphocyte; MO, monocyte; NE, neutrophil; PLT, platelet count; WBC, white blood cell.

^#^ absolute count.

^§^ start treatment with dashatinib.

## 3 DISCUSSION

The terminal phase of CML is usually transformed into acute myeloid leukemia (AML) and a few B acute lymphoblastic leukemia (B‐ALL), rarely involving T‐lineage in clinical practice. Up to now, only a limited number of CML cases with T‐cell blast (52 cases) were reported.^2^, ^4^, ^5^, ^6^, ^7^ We reviewed these documents including a case of admission to our hospital, which clinical materials were summarized as follows: 52 patients with a median age of 41.5 (12‐72 years), male:female = 38:14. Extramedullary involvement was more common (34/52). In this case, the women presented with multiple masses of the neck and immunophenotype showed BM had been affected. Owing to lack of FISH and pathological biopsy results of lymph nodes, we cannot prove exactly whether extramedullary had been blasted before bone marrow involvement. RT‐PCR results showed that 13 of 15 cases were M‐bcr (p210) type and 2 cases with m‐bcr (p190). It is generally believed that BCR/ABL fusion gene transcripts in CML are mainly M‐bcr (p210), while m‐bcr (p190) is dominant in Ph + ALL. The coexpression of p210 and p190 were reported rarely in B‐cell CML‐BC and Ph + T‐ALL,^2^, ^8^ but the T‐cell BC of CML has not been reported. Surprisingly, both p210 and p190 appeared simultaneously during the CML‐BC in our case. It has been confirmed p190 has stronger tyrosine kinase activity and transformation potential than p210. Studies showed that CML patients with the p190 transcript are more likely to present in BP initially and a tendency to progress to lymphoid blast.^9^, ^10^

Most blasting CML patients (about 70%) have additional chromosomal abnormalities, which were associated with worse effect for therapy, the adverse prognosis, blasting more easily, and shorter overall survival (OS).^11^, ^12^ The most common aberrations, such as +8, 2Ph, and i (17q), have been recognized as independent indicators of the progress of CML.^13^, ^14^ In some cases, translocations of genetic material between chromosome 11 and other chromosomes are associated with leukemia and lymphoma. At present, 17 chromosome 11p15 abnormalities associated with hematologic malignancies have been discovered, 15 of which involve the gene NUP98. The fusion genes of NUP98 gene and a large number of ‘partner genes’ are associated with many hematopoietic malignancies, such as MDS, AML, CML‐BC, and T‐ALL.^15^ Among these, NUP98‐RAP1GDS1 and NUP98‐ADD3 fusion genes resulting from t(4;11)(q21;p15) and t(10;11)(q11;p15), respectively, are most closely related to T‐ALL.^16^, ^17^ Reciprocal translocation of chromosomes 10 and 11 t(10;11)(q11;p15) was found in our case. Although the breakpoints of chromosome 10 in our case are different from previous report, 11p15 was involved, supporting the blastic transformation of CML to T‐line.

Chronic myelogenous leukemia with T‐cell blast crisis is easily confused with Ph + de novo T‐ALL/LL, especially when patients lack a history of CML or only involve extramedullary organs. It is of great practical significance to differentiate T‐cell blast crisis of CML from primary T‐lymphocyte disease timely and accurately, because of differences in treatment options and prognosis between the two entities. Based on traditionally clinical presentations, the former is frequent in adults and usually has a history of CML, extramedullary involvement alone, and without cell receptor rearrangement. Conversely, younger, lack of the natural course of CML, bone marrow involvement, and cell receptor gene rearrangements tend to be diagnosed as Ph + primary T‐LBL/ALL. Ichinohasama et al^11^ reported two easily confused cases of CML. FISH of lymph node showed that BCR‐ABL fusion gene was found in normal cells with normal morphology and karyotype but not in lymphoma cells, which proved that lymphoma and CML were coexisting instead of extramedullary involvement caused by CML. Thus, FISH technique combined with morphological examination is more reliable means of identification. Our case is an adult with CML history, T‐cell markers, M‐bcr and m‐bcr coexpressing, and t(10;11) all support the rapid conversion of CML to T‐ALL.

Currently, there is no standard treatment for the rapid progression of CML to T‐LBL/ALL. Most of the patients who received combined chemotherapy for T‐LBL/ALL died after several months of treatment. One case reported that imatinib was used to treat CML to T‐LBL/ALL with a remission period of more than 2 years.^18^ Notably, Imatinib resistance has been confirmed in patients with ABL kinase domain region mutations.^19^ For these patients, early hematopoietic stem cell transplantation (HSCT) may be a better choice. Two patients receiving HSCT had a overall survival period of up to 5 years based on reports.^6^, ^20^ Due to lack of large‐scale prospective study, the efficacy of HSCT for patients with CML‐BC remains to be further evaluated. Unfortunately, we cannot provide more useful information on treatment.

## 4 CONCLUSION

In summary, our report broadens the spectrum of T cell of CML‐BC and provides useful molecular evidence to support the presence of m‐bcr transcript, and chromosome 11p15 is crucial for T‐LBL/ALL from blast crisis of CML.

## AUTHOR CONTRIBUTIONS

Yuchao Yang and Weixiao Ren performed molecular analysis. Jianmei Chang and Li Guoxia performed cytogenetic analysis. Jianlan Li performed morphological analysis. Fanggang Ren performed flow cytometry analysis. Hongwei Wang helped revise the manuscript. Ting Xia and Zhifang Xu collected the case information and drafted the manuscript. All authors have reviewed and agreed upon the manuscript content.

## ETHICS STATEMENT

The authors have no ethical disclosures to declare.
